# Supplementary material for: Comprehensive Transcriptomic Profiling of m6A Modification in Age-Related Hearing Loss
Source: Biomolecules. 2023 Oct 18;13(10):1537. doi: 10.3390/biom13101537 (PMC10605720; doi:10.3390/biom13101537)

1. Merge image of METTL3 (70KDa)

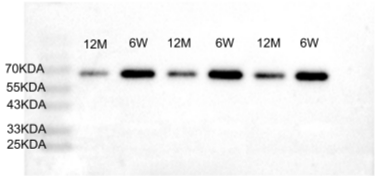

2. Merge image of FTO (58KDa)

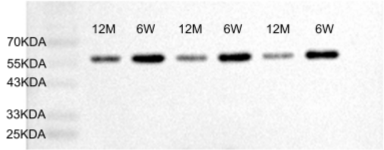

3. Merge image of WTAP (50KDa)

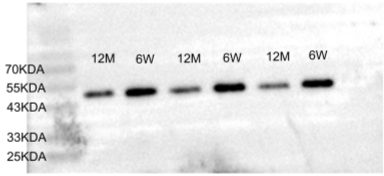

4. Merge image of ALKBH5 (40KDa)

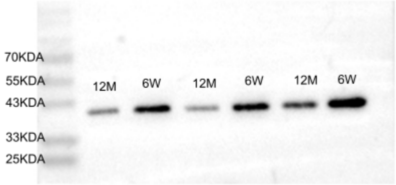

5. Merge image of GAPDH (36KDa)

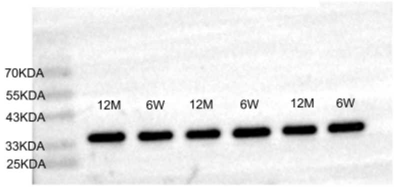

Supplement: Supplementary file 1 [file biomolecules-13-01537-s001.zip › biomolecules-2576250-supplementary.pdf]
